# Patient-derived xenograft models in hepatopancreatobiliary cancer

**DOI:** 10.1186/s12935-022-02454-9

**Published:** 2022-01-28

**Authors:** Binhua Pan, Xuyong Wei, Xiao Xu

**Affiliations:** 1 Department of Hepatobiliary and Pancreatic Surgery, The Center for Integrated Oncology and Precision Medicine, Affiliated Hangzhou First People’s Hospital, Zhejiang University School of Medicine, Hangzhou, 310006 China; 2 Zhejiang University Cancer Center, Hangzhou, 310058 China; 3 NHC Key Laboratory of Combined Multi-Organ Transplantation, Hangzhou, 310003 China; 4 Institute of Organ Transplantation, Zhejiang University, Hangzhou, 310003 China

**Keywords:** PDX model, Hepatopancreatobiliary cancer, Humanized

## Abstract

Animal models are crucial tools for evaluating the biological progress of human cancers and for the preclinical investigation of anticancer drugs and cancer prevention. Various animals are widely used in hepatopancreatobiliary cancer research, and mouse models are the most popular. Generally, genetic tools, graft transplantation, and chemical and physical measures are adopted to generate sundry mouse models of hepatopancreatobiliary cancer. Graft transplantation is commonly used to study tumour progression. Over the past few decades, subcutaneous or orthotopic cell-derived tumour xenograft models (CDX models) have been developed to simulate distinct tumours in patients. However, two major limitations exist in CDX models. One model poorly simulates the microenvironment of tumours in humans, such as the vascular, lymphatic and immune environments. The other model loses genetic heterogeneity compared with the corresponding primary tumour. Increased efforts have focused on developing better models for hepatopancreatobiliary cancer research. Hepatopancreatobiliary cancer is considered a tumour with high molecular heterogeneity, making precision medicine challenging in cancer treatment. Developing a new animal model that can better mimic tumour tissue and more accurately predict the efficacy of anticancer treatments is urgent. For the past several years, the patient-derived xenograft model (PDX model) has emerged as a promising tool for translational research. It can retain the genetic and histological stability of their originating tumour at limited passages and shed light on precision cancer medicine. In this review, we summarize the methodology, advantages/disadvantages and applications of PDX models in hepatopancreatobiliary cancer research.

## Introduction

Mouse models are critical tools in preclinical and translational research in hepatopancreatobiliary cancer [1], including drug screening, assessment of therapeutic efficacy, identification of biomarkers, and molecular subtyping. Traditionally, immortalized cancer cell lines derived from patient tumours are used to construct mouse models to simulate tumour growth in the human body [2]. In these models, immortalized cancer cell lines are injected into immunodeficient mice subcutaneously or orthotopically [3]. Comparatively speaking, subcutaneous tumour models cost little and are easy to construct and measure when the tumour grows; however, they cannot simulate the tumour environment in vivo [4]. Orthotopic tumour models can mimic the tumour environment to some degree but are challenging to construct and measuring the tumour size [4]. Both models have contributed substantially to the development of hepatopancreatobiliary cancer research in recent years. However, many investigators have shown that cell-derived xenograft models (CDX models) cannot accurately mimic the tumour condition in human genetic heterogeneity and the tumour microenvironment. Thus, more realistic and clinically relevant models are required to meet the higher remands of preclinical research.

In this context, patient-derived xenograft models (PDX models) have been developed to overcome these disadvantages of CDX models [5]. The PDX model was first reported in 1969. Rygaard and Povlsen implanted a piece of tumour from a 71-year-old patient with sigmoid cancer in nude mice that was maintained for 76 passages [6]. In 1996, the first PDX model of hepatocellular carcinoma was reported [7].

In recent years, PDX models have been verified to maintain the hereditary stability of primary tumours, at least at early passages [8]. Similar results have been reported in various cancers, such as diffuse large B-cell lymphoma (DLBCL) [9], breast cancer (BC) [10], non-small cell lung cancer (NSCLC) [11], neuroblastoma [12] and others [13].

In hepatocellular carcinoma (HCC) and pancreatic ductal adenocarcinoma (PDAC), Raquel et al. compared the transcription profile of PDX models in the 5th and 10th passages and found no major functional changes in the later passages [14]. Additionally, because the proportion of liver cancer in children is different from that in adults, orthotopic PDX models for paediatric liver cancer were also established [15]. The results indicated that the gene expression profiles for five generations (F0, F1, F2, F13, F20) appeared remarkably similar, and PDX models recaptured the metastatic ability of the primary tumour.

Therefore, PDX models are currently considered promising tools for clinical translational research in hepatopancreatobiliary cancer.

## Methodology of establishing PDX models

To develop a PDX model, tumour tissues from a patient were collected and transplanted into immunodeficient mice [8]. The passage harbouring tumour tissue is called F_0_ (or G_0_), and successful passages are named consecutively (F_1_, F_2_, F_3,_ etc.) [8]. Usually, tumours obtained in surgical operations or biopsy are used to develop PDX models. Additionally, tumour cells acquired from ascites [16] or pleural effusion [17] are reported to be valid in developing PDX models in some tumour types. For tumour fragments, two different pretreatment methods are used before implantation. Investigators cut the tumour tissue into approximately 20–50 mm^3^ pieces [15, 18], followed by subcutaneous or orthotopic implantation. Others prepare tumour tissues into a single-cell suspension for subsequent implantation [19–21]. Both methods have advantages and disadvantages. Tissue fragments can retain cell–cell interactions, which mimic the tumour microenvironment, and single-cell suspensions can avoid heterogeneity inside the tumour to some degree but have a lower success rate because of chemical or physical damage during pretreatment [22]. In most studies, fresh tumour tissue is selected for subsequent implantation. Frozen tumour tissue, however, is also reported in some studies to be an efficient source for implantation [10].

The success rate of developing PDX models is also associated with the mouse strain used to implant tumour tissue. Nude, NOD-SCID and NSG mice are commonly used to develop PDX models. The nude mouse is characterized by the absence of a thymus, resulting in a substantially reduced number of T cells. Additionally, body hair is lacking, making it easy to measure the tumour size. However, functional B cells and NK cells are retained in the body of nude mice, making it challenging for primary human tumour tissue to grow. Thus, the success rate of PDX models developed in nude mice is undesirable. NOD-SCID mice and NSG mice both have more severe immunodeficiency than nude mice because they have no functional T cells or B cells. Additionally, NSG mice have a complete deficiency of NK cells compared with NOD-SCID mice. Therefore, the NSG mouse was recently regarded as the most effective mouse strain for developing PDX models. Theoretically, the optimal transplantation site is exactly where the primary tumour grows, termed orthotopic implantation. Compared with subcutaneous implantation, it offers the most similar anatomic environment, retains spontaneous metastatic capacity [23] and has a higher engraftment rate in some tumour types [24]. However, only a small percentage of studies [15, 25] are based on orthotopic implantation because of its higher requirement for surgical technology and steep cost. Additionally, unlike subcutaneous implantation, imaging technology is needed to monitor tumour growth. The most common implantation site selected is the subcutaneous region in the flanks. This site is not complicated to evaluate, tumour growth can be easily monitored, and the procedure is inexpensive. More than 1000 subcutaneous PDX models of different tumour types, 195 of which were hepatopancreatobiliary cancer, were built by Novartis Institutes, contributing considerably to drug screening [26]. The renal capsule is also a good choice for implantation; it was reported to have a high engraftment rate (36 succeeded in 36) [27] in prostate cancer and to shorten the time for implantation. However, no similar studies have been performed in liver cancer thus far. At our centre, more than 100 HCC tissues have been implanted, and the uptake rate is approximately 39.47% (30/76) [28].

## Applications of PDX models in hepatopancreatobiliary cancer

Hepatopancreatobiliary cancer, including liver cancer, pancreatic cancer, and biliary cancer, is characterized by high lethality and poor survival. Surgical resection is the most effective treatment for hepatopancreatobiliary cancer. However, many patients do not have an opportunity for surgery when the tumour is detected [29]. Because of its high heterogeneity, patient responses to other treatments vary widely [30]. Therefore, strategies to guide precision medicine. The most widely used animal models for hepatopancreatobiliary cancer are established using cell lines, which cannot simulate the heterogeneity of tumour tissues. PDX models may overcome this drawback. In hepatopancreatobiliary cancer, PDX models maintained gene expression similarities with primary tumours, and no major functional changes occurred between F5 and F10 PDX passages [14]. Recently, many studies have shown that the response rates to treatment in PDX models are highly consistent with those in patients [4, 31–35]. Therefore, PDX models are better preclinical for precision medicine in hepatopancreatobiliary cancer.

## Precision selection of antitumour drugs

Cancer cell lines have been essential tools in drug screening for more than 25 years. The US National Cancer Institute (NCI) has developed 60 human cancer cell lines (NCI-60) to meet the demands [36]. However, in the spring of 2016, the institute retired the NCI-60 and launched a new cancer model derived from fresh patient tumour tissues instead. Cell lines were retired because they have been cultured for thousands of generations, making them adapt to survive on plastic culture disks and differ greatly from primary tumour tissues in genetic make-up and behaviour. Compared with cell lines, fresh tumour tissue shares the same genetic profile as the human body, and investigators have shown that PDX models maintain most genetic features compared to primary human tumour tissue.

For pancreatic ductal adenocarcinoma (PDAC), the response to gemcitabine in PDX models showed a strong correlation with that in clinical patients [37]. In hepatocellular carcinoma, sorafenib (HCC), an oral multikinase inhibitor, is the only US Food and Drug Administration (FDA)-approved first-line systemic therapy until September 2018, when lenvatinib was also approved by the FDA. The efficacy of sorafenib has been tested in the HCC PDX model, which was revealed as a promising tool to predict the efficacy of sorafenib because it shows a response to treatment similar to that in the primary patient [18, 38].

Additionally, PDX models were used to develop new therapeutic strategies in sorafenib-resistant patients. Mark Kin found that stearoyl-CoA desaturase-1 (SCD1) regulated sorafenib sensitivity through endoplasmic reticulum (ER) stress and that using an SCD1 inhibitor combined with sorafenib showed a suppressive effect in a sorafenib-resistant PDX model [39–41]. They also revealed that CD47 upregulation was associated with sorafenib resistance and that the combined use of an anti-CD47 antibody with sorafenib reversed drug resistance [42]. Other drug combination therapy strategies, such as MEK inhibitors combined with sorafenib [43] and mTOR inhibitors combined with bevacizumab [44], have been tested in HCC PDX models and have shown a significant suppressive effect on tumours [45–47].

Novartis [26] established 195 PDX models of hepatopancreatobiliary cancer containing different driver mutations to assess the population responses to diverse treatments. The response of PDX models to different treatments was similar to data collected from clinical trials. Thus, the PDX model has potential application value in the preclinical evaluation of cancer treatment drugs or methods and can predict the clinical trial response to some extent.

However, one major limitation of the PDX model is the loss of human tumour stroma, which is completely replaced by murine stroma in the second generation [48]. Because the interaction between tumour cells and the microenvironment plays a critical role in tumour progression, it likely affects the response of PDX models to a certain treatment. To overcome this problem, coimplantation of human stromal cells with primary tumour tissue may optimize traditional PDX models.

## PDX models in identifying tumour biomarkers for molecular subtyping

Clarifying the consistency between the PDX model and primary tumour has not only helped to predict the effectiveness of drugs but has also helped to identify molecular biomarkers related to drug sensitivity or resistance [4] and patient prognosis [49, 50].

In HCC patients, frequent mutations of tuberous sclerosis complex (TSC1) and TSC2 were detected (16.2%, 18/111) [51]. Compared with normal PDX models, TSC2-mutated PDX models appeared more sensitive to rapamycin, an mTOR inhibitor, suggesting that individuals with different TSC expression levels had different susceptibilities to rapamycin. Intriguingly, another research group found that TSC2 was negatively correlated with the efficacy of sorafenib. In PDX models TSC2 upregulation, sorafenib treatment aggravated HCC progression [52], indicating that the PDX model is a promising tool to guide clinical medication. Additionally, polymeric immunoglobulin receptor (pIgR) was recently reported to promote cell transformation and proliferation, contributing to tumour growth. In PDX models, pIgR was shown to be an effective biomarker to predict the efficacy of dasatinib and MEK inhibitors [53].

In another study with 60 PDX models, whole-exome sequencing (WES) was performed on third-generation tumours [54]. Four PDX models were confirmed to contain JAK1 mutations. Compared with other normal models, these four PDX models were sensitive to ruxolitinib, an inhibitor of JAK-STAT, suggesting that PDX models effectively promote the development of HCC therapies. Other biomarkers, such as acetyl-coenzyme A carboxylase alpha (ACCα) [55], CD133 and CD44 [56], were also reported to be associated with tumour growth and a worse prognosis in PDX models.

In conclusion, the PDX model is biologically stable and maintains the gene profile of the primary tumour. Predicting sensitivity or resistance by identifying biomarkers is critical to help develop precision medicine strategies for patients with similar gene expression.

## PDX models and Coclinical trials

Tumours are heterogeneous diseases, and individuals’ responses vary with the same treatment. Therefore, a valid model that better links basic research and the clinic is required to overcome this obstacle. The major problems of the traditional mouse model are the lack of tumour heterogeneity and genetic diversity between the models and primary tumours [57]. Medicine proved to be efficient in a traditional animal model [mainly cell-line-derived xenograft models (CDX) models] and had a low response rate when used in clinical trials. More than 95% percent of novel therapies fail in clinical trials [58]. Additionally, when a drug enters clinical trials, analysing and integrating information that may help to prioritize drug use are challenging [4].

Based on this background, the “coclinical trial” project was initiated by Caterina Nardella et al. [59]. They exploited mouse models that faithfully simulate the mutations observed in human bodies to perform preclinical trials parallel to ongoing human clinical trials. The mouse model they used was genetically engineered mice (GEM), and the PDX model was also used by investigators in other parallel studies called “Mouse Avatar” [57, 60]. In coclinical trials, anticancer reagents were administered to patients with a defined genetic makeup and mouse models with similar genetic mutations [60]. Generally, the aim of this project was to optimize treatment strategies in clinical trials to identify the best treatment strategy for patients.

The project was supported by several studies in pancreatic cancer and other tumour types [61–63]. Several clinical trials are ongoing to validate the efficiency of Mouse Avatar. In NCT02795650 [64], patients with metastatic pancreatic adenocarcinoma (PDAC) were recruited, and PDX models were developed to evaluate personalized treatments. Unfortunately, no similar coclinical trials are in progress in HCC that remain to be performed.

In summary, although more evidence must be collected, a coclinical trial is a promising model to optimize therapeutic strategies for patients with tumours. With these Avatar models, therapeutic regimens could be adjusted in a timely manner for a better response instead of waiting for clinical outcomes in patients.

## Mini-PDX models in cancer therapy

Although PDX models are cracking tools to evaluate anticancer drug responses, the shortcomings of PDX models are also very obvious. In addition to its high cost, the largest obstacle is that it usually takes 2–6 months for the tumour to grow. This delayed feedback from PDX models limits their application in guiding the treatment of the original patients. To overcome this obstacle, a rapid in vivo drug sensitivity assay called mini-PDX was developed that can test the response of antitumour cancer drugs within 7 days [65].

To establish a mini-PDX model, tumour tissue is washed with Hank’s balanced salt solution to wipe off nontumour tissue followed by digestion with collagenase. After the preparation of the cancer cell suspension, it is filled into a modified microencapsulation and hollow fibre culture system (OncoVee capsules). Next, the capsules are transplanted subcutaneously into immunodeficient mice, and the mice are subsequently treated with antitumour drugs. Therapeutic responses are evaluated by measuring tumour cell proliferation in the capsules.

In a recent study, 26 tumour tissues acquired from patients with pancreatic cancer and other tumour types were collected to establish mini-PDX models. The efficiency of S-1, docetaxel, oxaliplatin, irinotecan and other drugs was assessed in mini-PDX models. Afterwards, the therapeutic response was compared with known clinical outcomes in patients. The mini-PDX model had a sensitivity of 80% and a specificity of 93%, indicating that the mini-PDX model is a powerful tool to evaluate efficiency [65]. In a case of pancreatic cancer, the sensitivity of chemotherapy drugs was consistent with the clinical response in the original patient. The patient, treated with the therapeutic regimen evaluated by the mini-PDX model, had a good reaction [66]. In another study, 12 gallbladder carcinoma tumour tissues were used to establish mini-PDX models to examine the sensitivity of five chemotherapy drugs. The results were adopted to guide the treatment of patients after surgery. Compared with patients treated with traditional chemotherapy, patients in the mini-PDX guided therapy group had a longer overall survival (OS) (18.6 months vs. 13.9 months) and longer disease-free survival (DFS) (17.6 months vs. 12.0 months) [67].

In conclusion, mini-PDX models are suitable for selecting effective or elucidating noneffective therapeutic strategies and show promise in precision medicine.

## Humanized PDX models in cancer immunotherapy

The immune system plays a crucial role in both promoting and inhibiting tumour growth [68], and immunotherapy is a promising tool in cancer treatment. However, xenograft models are built on immunocompromised mice to avoid rejection of transplanted human tumour tissue or cells. Thus, these models are invalid in investigating the tumour microenvironment (TME), including the infiltration of immune cells and crosstalk between the tumour and immune system. To overcome these challenges, several humanized mice were developed, including genetically engineered humanized mice and immunologically humanized mice [69]. Genetically engineered humanized mice were developed by replacing the murine gene with an equivalent human transgene [70], and immunologically humanized mice were generated by engraftment of human immune cells. Thus, immunologically humanized mice can be used to construct PDX models.

CD34^+^ human haematopoietic stem and progenitor cells (HPSCs) are engrafted into immunodeficient mice to reconstruct the human immune system. HPSCs are isolated from human foetal liver samples or from cord blood by density gradient centrifugation [71]. The efficiency of reconstitution was evaluated by [%hCD45 + /(%hCD45^+^ + %mCD45^+^)], and recipient mice with 20–50% human CD45^+^ cells were used for subsequent implantation (HPSC-PDX) [72]. In this model, the human immune system, including functional T cells, natural killer cells and monocytes, can be developed [73].

In other studies, immunodeficient mice were transplanted with human peripheral blood mononuclear cells (PBMCs) followed by engraftment of tumour tissue to develop a humanized PDX model (PBMC-PDX) [74, 75]. Compared with HPSC-PDX, PBMC-PDX has a lower time cost (4 weeks vs. 10–14 weeks) and is more accurate in evaluating PD-L1/PD-1 targeted immunotherapies [75]. However, the time window for research in PBMC-PDX is only 3–4 weeks because of severe graft-versus-host disease (GVHD) [70, 76].

## Conclusions

Patient-derived xenograft models (PDX models) have attracted increased attention in preclinical cancer research over the past several years. They play crucial roles in evaluating the efficiency of antineoplastic drugs and screening for biomarkers of drug sensitivity and resistance. To overcome the challenges of conventional PDX models, such as the lack of a tumour microenvironment (TME) and long-term cost, humanized PDX models have been developed to mimic the human immune system, and mini-PDX models have been developed to evaluate drug efficiency in a faster approach. A new project called “coclinical trials” was proposed to use PDX models as mouse avatars to optimize antitumour treatment strategies. These efforts were made to improve the survival rate of patients with tumours. Although PDX models have many advantages compared with traditional CDX models and are increasingly widely used in different stages of cancer research, shortcomings remain to be solved. One major limitation of the PDX model is the loss of human tumour stroma. Another is that the pharmacokinetic properties of drugs differ in diverse species, making it difficult to predict the efficacy of drugs in patients by animal models.

**Fig. 1:**
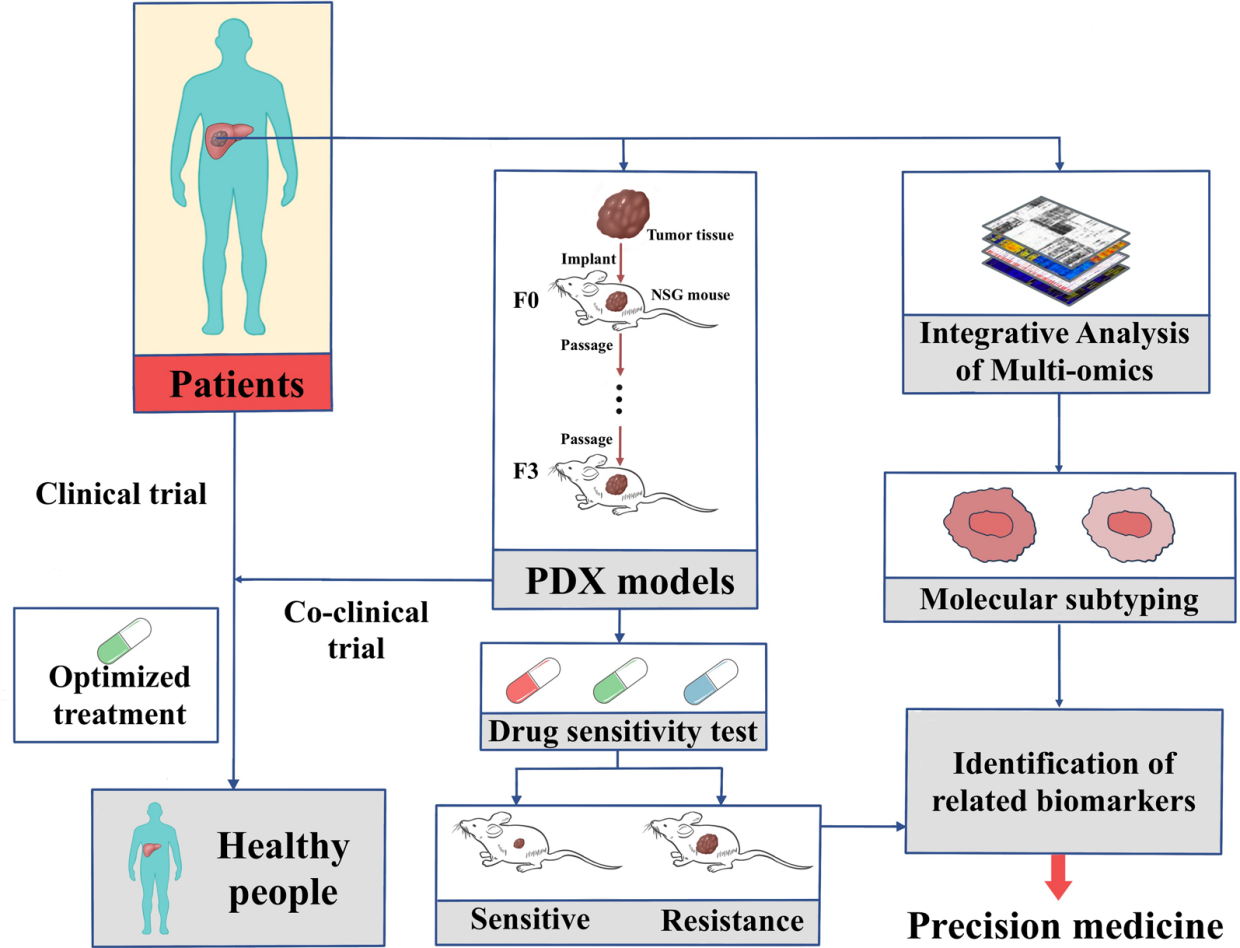
Methodology of establishing PDX model and applications. Tumour tissues collected from patients were transplanted into immunodeficient mice, 3rd passage PDX model was usually used for testing drug efficacies and co-clinical trials

**Fig. 2:**
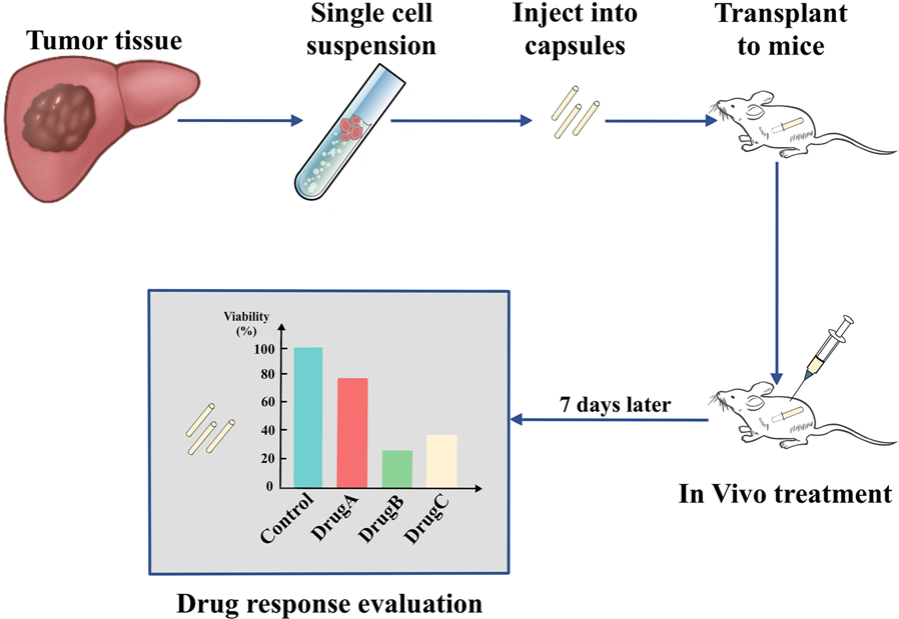
Methods to establish a Mini-PDX model. Single tumour cell suspension were prepared before injected into capsules, the capsules were then transplanted to mice for in vivo treatment and subsequent evaluation

In conclusion, the PDX model will play an increasingly important role in the individual treatment of patients with cancer (Figs. 1, 2).

## Data Availability

The material supporting the conclusion of this review has been included within the article.
